# An epistatic effect of *apaf*-*1* and *caspase*-*9* on chlamydial infection

**DOI:** 10.1007/s10495-015-1161-x

**Published:** 2015-08-20

**Authors:** Mohd. Akhlakur Rahman, Mutsunori Shirai, Md. Abdul Aziz, Rie Ushirokita, Sayuri Kubota, Harumi Suzuki, Yoshinao Azuma

**Affiliations:** Department of Microbiology and Immunology, Yamaguchi University School of Medicine, 1-1-1, Minamikogushi, Ube, Yamaguchi 755-8505 Japan; Biology-Oriented Science and Technology, Kindai University, Nishimitani 930, Kinokawa, Wakayama 649-6493 Japan

**Keywords:** *Chlamydia pneumoniae*, Apaf-1, Caspase-9, Apoptosis, Infection, *Chlamydia trachomatis*, Host–parasite interaction

## Abstract

*Chlamydia* is an obligate intracellular bacterial pathogen that replicates solely within a membrane-bound vacuole termed an inclusion. *Chlamydia* seems to perturb multiple cellular processes of the host, such as, rearrangement of the membrane trafficking system for its intracellular multiplication, and inhibition of host cell apoptosis for persistent infection. In an attempt to clarify host factor involvement in apoptosis regulation, we found that inhibition of Caspase-9 restricted, while Apaf-1 promoted, *Chlamydia pneumoniae* infection in HEp-2, HeLa, and mouse epithelial fibroblast (MEF) cells. These opposition contributions to the chlamydial infection were confirmed using *caspase*-*9*^−/−^ and *apaf*-*1*^−/−^ MEFs. Similar phenomena also appeared in the case of infection with *Chlamydia trachomatis*. Interestingly, caspase-9 in *apaf*-*1*^−/−^ MEFs was activated by chlamydial infection but during the infection caspase-3 was not activated. That is, caspase-9 was activated without support for multiplication and activation by Apaf-1, and the activated caspase-9 may be physically disconnected from the caspase cascade. This may be partially explained by the observation of caspase-9 accumulation within chlamydial inclusions. The sequestration of caspase-9 by chlamydia seems to result in apoptosis repression, which is crucial for the chlamydial development cycle. Because Apaf-1 shares domains with intracellular innate immune receptor NOD1, it may play a key role in the strategy to regulate chlamydial infection.

## Introduction

Apoptosis or programmed cell death is an active process of cellular suicide triggered by a variety of stresses and physiological stimuli for tissue development and homeostasis of organisms [1]. The morphological and biochemical features of apoptosis are caused by activation of caspase cascades in both of the major pathways, the death receptor-mediated extrinsic pathway and the mitochondria-mediated intrinsic pathway [2]. In the intrinsic pathway, apoptosis is initiated by Bax-dependent cytochrome *c* release from mitochondria, followed by apoptosome formation with cytochrome *c*, AMP, Apaf-1, and pro-caspase-9 [3]. Caspase-9 activated within the apoptosome promotes caspase-3 activation leading to the apoptotic nuclear structure [4]. This apoptotic response is tightly regulated by Bcl-2 to prevent the release of cytochrome *c* from mitochondria [5].

Elimination of pathogen-infected cells is an important role of apoptosis as a primary defense against viruses and intracellular bacteria by precluding metabolic support for pathogen multiplication and engaging competent professional phagocytes for the action of immune defense mechanisms [6]. Coping with defensive systems of host organisms as growth environments, intracellular bacteria, such as *Shigella* and *Legionella*, are thought to have developed a variety of strategies for modulating the apoptotic process in host cells, especially during early stages of the life cycle [7].

*Chlamydia pneumoniae* is an obligatory intracellular bacterium that was first described in 1986 as a pathogen for acute respiratory diseases [8]. It has also been implicated in the onset or progression of several chronic diseases including asthma [9], atherosclerosis [10] and Alzheimer’s disease [11]. As a refined strategy to facilitate their survival and evade the host immunosurveillance, *Chlamydia* modulates host-cell pathways including apoptosis regulation. But the action of *Chlamydia* on host apoptosis has been successively reported with different outcomes from repression to promotion, mainly ascribed to the different conditions, different combinations of chlamydial species and host cell types, and different stages of infection [12]. *Chlamydia psittaci* and *Chlamydia trachomatis* have been reported to induce apoptosis of cultured cells [13–16], but *C. trachomatis* has also been shown to repress CD95-induced apoptosis [17]. While *C. pneumoniae* was reported to inhibit apoptosis induced by staurosporine (STS) and tumor necrosis factor alpha (TNF-α) in infected epithelial cells, macrophages and monocytes [18–21]. However, it was reported that *C. pneumoniae* induced apoptosis in coronary artery endothelial cells [22], whereas in many cases *C. pneumoniae* tends to suppress host apoptosis. Elucidation of host cell apoptosis controlled by *Chlamydia* is a prerequisite to understanding chlamydial strategies for persistent infection and how to overcome the diseases caused by *Chlamydia*.

The involvements of many host and chlamydial factors have been described for inhibition of host apoptosis by *Chlamydia* species. The first installment reported that host cell apoptosis promoted by a variety of stimuli, such as STS and TNF-α, was inhibited by chlamydial infection. This inhibition was accompanied with and explained by prevention of the cytochrome *c* release from mitochondria [21, 23]. This prevention was later explained by specific degradation of the pro-apoptotic BH3-only proteins, such as Bik, Puma, Bim, Bad, Bmf, Noxa, and tBid [24–26]. Chlamydial protease, or proteasome-like activity factor (CPAF), which is a potent and promiscuous cysteine protease capable of cleaving many host proteins, was initially implicated in this degradation [27]. However, subsequent studies showed that the proteolysis of the reported CPAF substrates was due to enzymatic activity in cell lysates rather than in intact cells [28, 29]. Moreover, conflicting observations concerning the degradation of the pro-apoptotic BH3-only proteins were also reported [30, 31]. Thus, the involvement of BH3-only proteins and CPAF is still an important topic to be clarified.

Instead of the degradation of pro-apoptotic factors, stabilization of the anti-apoptotic factor Bcl-2 has been described [31, 32]. Along with protection from host cell apoptosis during *C. trachomatis* infection, the activation of both Raf/MEK/ERK (or MAPK/ERK) and PI3K/AKT pathways has been observed, leading to up regulation of *mcl*-*1* gene expression and stabilization of Mcl-1, which binds to the BH3-only protein, Bim and inhibits apoptosis initiation [31]. Recently, Bcl-2-associated athanogene (Bag-1), which interacts with a diverse array of molecular targets including anti-apoptotic regulator Bcl-2 and heat shock proteins, was identified as another element that is potentially regulated via the MAPK/ERK pathway [32].

Two interesting sequestration models have been proposed, based on evidence suggesting that pro-apoptotic factors are mislocalized away from their conventional target sites in infected cells. In the first study, activation of the PI3K pathway by *C. trachomatis* infection, but not *C. pneumoniae*, led to phosphorylation of Bad, and the phosphorylated Bad was sequestered via 14-3-3 beta in the chlamydial inclusion membrane that expresses IncG proteins [30]. The other observation was that protein kinase C delta (PKC-δ), which functions as a pro-apoptotic effector in the mitochondria and nucleus, was mislocalized in the immediate vicinity of chlamydial inclusions where diacylglycerol was accumulated [33]. In both cases, it was not mentioned whether those factors work only to trigger apoptotic regulation or serve any other special functions at the sequestration sites.

The engagement of downstream molecules has also been suggested. *Chlamydia pneumoniae* infection of human monocytic cells induced the expression of the cellular inhibitor of apoptosis 2 (cIAP2) by misuse of the NF-κB pathway during infection [34]. Infection with *C. trachomatis* also led to the up regulation of cIAP2 and stabilized functional heterodimers of the IAPs, thereby the ability to inhibit apoptosis may be more secure [35].

Herein we report that an epistatic effect of the apoptosome components *apaf*-*1* and *caspase*-*9* on chlamydial infection and its host apoptosis regulation; that is, caspase-9 functions as in the sequestration model of pro-apoptotic factors for the regulation of host apoptosis, and Apaf-1 restricts chlamydial infection. Interestingly, *Chlamydia* required caspase-9 activation for its infection, but this did not lead to caspase-3 activation or apoptotic events, and caspase-9 activation occured without Apaf-1. Our results suggest that the ratio of Apaf-1 and caspase-9 influences host susceptibility to chlamydial infection.

## Materials and methods

### Reagents and antibodies

Apoptosis inhibitors, Hoechst 33258, 4′-6-diamidino-2-phenylindole (DAPI), and cell-permeant inhibitors of Apaf-1 (NS3694), caspase-8 (Z-IETD-FMK), caspase-9 (Z-LEHD-FMK), and caspase-3 (Z-EDVD-FMK) were obtained from Sigma-Aldrich (Saint Louis, MO). Fetal calf serum was from Cansera International Inc. (Etobicoke, Canada). STS, gentamicin, penicillin, streptomycin, and cycloheximide were from Wako (Tokyo, Japan). Anti-Apaf-1, anti-caspase-9 and anti-caspase-3 antibodies were from Cell Signaling Technology (Danvers, MA). Anti-caspase-9 antibodies were also purchased from Calbiochem (La Jolla, CA) and Abcam (Cambridge, UK). Caspase-9, -8 and -3 Colorimetric Activity Assay kits, and ApopTag Fluorescein kit for TUNEL assays were from Chemicon (Temecula, CA). Chlamydiaceae-specific fluorescein isothiocyanate (FITC)-conjugated monoclonal antibody (Chlamydia-FA) was from Denka Seiken (Tokyo, Japan).

### Host cell lines, chlamydial strains and infection

Apaf-1 knockout (*apaf*-*1*^−*/*−^) and caspase-9 knockout (*caspase*-*9*^−*/*−^) mouse epithelial fibroblasts (MEF), and Bcl-2-overexpressing HeLa cells were kind gifts from Xiaodong Wang (Univ of Texas) [36], Shin Yonehara (Kyoto Univ) [37] and Yoshihide Tsujimoto (Osaka Univ) [3], respectively. These cell lines and their corresponding control cells, i.e., MEFs, HeLa229 (ATCC CCL-2), and HEp2 (ATCC CCL23) were cultured in Dulbecco’s modified Eagle’s medium supplemented with 2 mM l-glutamine (Sigma-Aldrich), 10 % heat-inactivated fetal calf serum and 50 µg/mL gentamicin at 37 °C under 5 % CO_2_. *Chlamydia pneumoniae* J138 and AR39, and *C. trachomatis* serovar D were used for chlamydial infection.

Host cells, 2 × 10^4^ cells per well of flat-bottomed 96-well tissue culture plates, were allowed to adhere for 24 h prior to infection. Measurements of infection rates for *C. pneumoniae* J138 were calculated by the same method described previously [38], or as described in the figure legend for each experiment. Briefly, the multiplicity of infection (MOI) of each chlamydial stock solution was first calculated and determined by its inclusion formation units (IFUs) against HEp-2 cells. Infection rates achieved at MOI = 0.2 in HEp-2, HeLa, and MEFs were approximately from 15 to 25 % in our experiments. Infection was generally carried out at MOI = 0.2 to given host cells. After cells were fixed at 48 h post-infection (hpi) and stained with Chlamydia-FA and DAPI, cells with inclusions only larger than 4 µm in diameter were counted as infected ones, to adjust the infectious stage and diminish staining noises. Infection rates were calculated based on cell numbers determined by DAPI staining of nuclei. Generally more than 100 infected cells were counted as a population for one sample. All data are expressed as mean ± SD calculated from at least three independent experiments. An asterisk denotes *p* < 0.05 with Student’s *t* test.

Amounts of infectious progenies of *C. pneumoniae* were calculated as previously described [38]. Briefly, culture supernatants of chlamydia infection at 80 hpi were harvested and used for re-infection in control MEFs. The infection rates were measured at 48 hpi.

### Apoptosis induction and assays

HEp-2 cells were infected with *C. pneumonia* J138 (MOI = 0.2). At certain times during infection, apoptosis of host cells was induced with 0.5 μM STS for 4 h. After fixation with 30 % and then 70 % ethanol for 10 min at room temperature, cells were stained with Chlamydia-FA and 2 µm Hoechst 33258 in phosphate-buffered saline (PBS) for 45 min at 4 °C.

For the categorization of apoptotic or non-apoptotic cells in the infection cases, only cells containing inclusions larger than 4 µm in diameter were counted as the general population of infected cells. Cells with smaller inclusions or without any inclusions were eliminated from the cell counting. Out of more than 50 infected cells, which were selected randomly under 200 times magnification, cells showing apoptotic nuclear morphology were counted. All data are expressed as mean ± SD calculated from at least three independent experiments. An asterisk denotes *p* < 0.05 with Student’s *t* test. To confirm apoptotic cell death, we carried out TUNEL staining as described previously [33].

For caspase-8, -3 and -9 activity assays, cytosolic extracts were prepared and analyzed by Colorimetric Activity Assay Kit (Chemicon), according to the manufacturer’s protocol. For western blot detection of Apaf-1, caspase-3 and -9 protein, total cell extracts were prepared from *apaf*-*1*^−*/*−^ and control MEFs in the lysis buffer of the caspase activity assay kit, and western blotting assays to detect caspase cleavage were performed as described previously [39].

### Immunofluorescence staining

For analysis of the localization of caspase-9, host cells grown on coverglasses (with or without chlamydial infection) were fixed with 100 % methanol and independently stained with anti-caspase-9 antibodies purchased from Cell Signaling, Calbiochem and Abcam. To detect chlamydial inclusions, mouse anti-*Chlamydia* sp monoclonal antibody RR402 (Washington Research, Seattle, WA) and rhodamine-conjugated goat anti-mouse antibody (DAKO) were used. Polyclonal antibody against inclusion membrane protein A2 (IncA2) of *C. pneumoniae* J138 was produced using recombinant IncA2 protein by the same method as described previously [39].

### Vector construction and transfection

pCMV-SPORT6.1 containing the mouse *apaf*-*1* gene was obtained from Invitrogen (Carlsbad, CA). The pCMV control vector was prepared by removing the 0.9-kb *Hind*III—*Hind*III region containing the mouse *apaf*-*1* gene from the pCMV-SPORT6.1. Transfection was carried out with a Nucleofector (Lonza, Cologne, Germany) based on the manufacturer’s recommended methods. The infection assays were carried out when the rates of the transient transfection were above 70 %.

## Results

### Apoptosis repression by *Chlamydia pneumoniae*

**Fig. 1:**
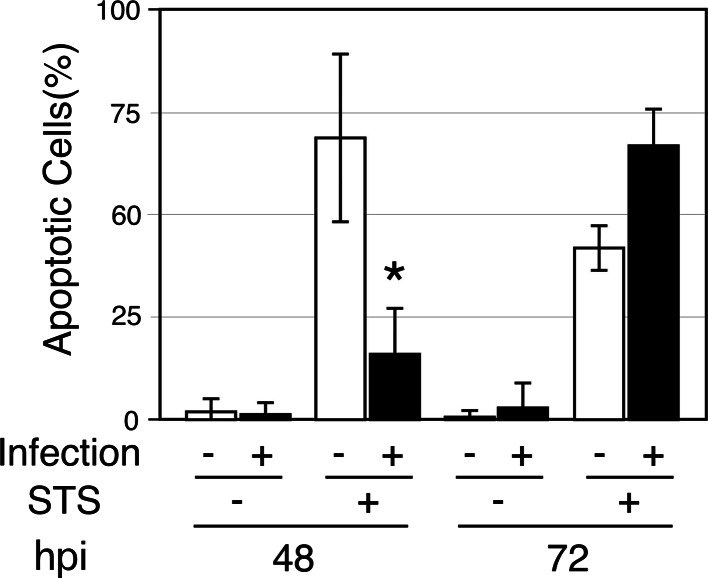
*Chlamydophila* *pneumoniae* regulates host apoptosis under apoptotic stimulation using STS. HEp-2 cells were infected with *C. pneumonia* J138 (MOI = 0.2), and STS (final concentration, 0.5 µm) was added at 44 and 68 h post-infection (hpi). Four hours later (shown as 48 and 72 hpi, respectively), cells were stained with Hoechst 33258. Only cells containing inclusions larger than 4 µm in diameter were counted as infected cells, and the infected cells were categorized into either apoptotic or non-apoptotic cells. Cells with smaller inclusions or without any inclusions were eliminated from the cell counting. All data are expressed as mean ± SD calculated from at least three independent experiments. An *asterisk* denotes *p* < 0.05 using Student’s *t* test

We verified the involvement of *C. pneumoniae* J138 in HEp-2 cell apoptosis. *Chlamydia pneumoniae*-mediated blockage of STS-induced apoptosis was found at 48 hpi and this blockage ceased by 72 hpi (Fig. 1). Similar responses were observed using HeLa cells and MEFs (data not shown). In the absence of apoptotic stimuli, apoptotic induction by *C. pneumoniae* infection was not observed between 48 and 72 hpi, which are the middle and late stages of *C. pneumoniae* infection, respectively [40]; chlamydial infection partially stimulated STS-induced apoptosis at 72 hpi. These data, combined with previous results [18–21], indicate that *C. pneumoniae* infection at relatively low MOI represses STS-induced apoptosis of various host cell lines in the early-to-middle stages of infection, but not in the late stage.

### Preference of anti-apoptotic environments for chlamydial infection

**Fig. 2:**
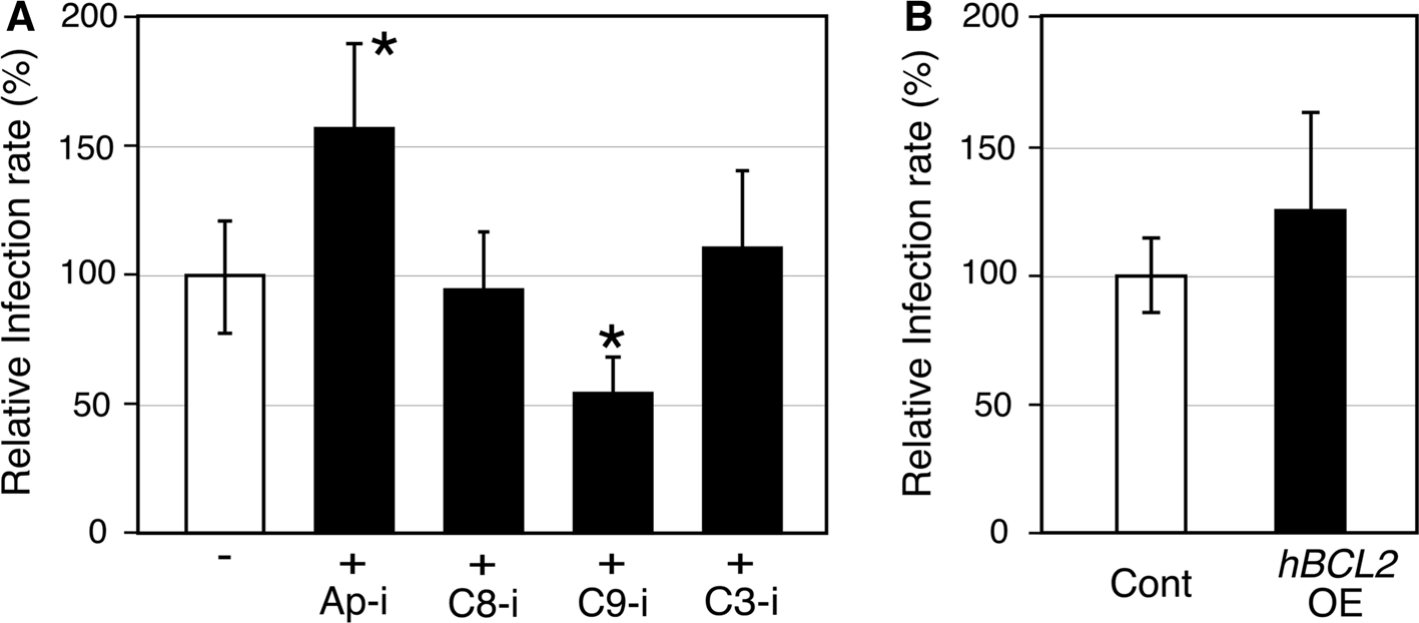
Apaf-1 and caspase-9 inhibitors show opposing contributions to *C. pneumonia* infection. **a** HEp-2 cells treated with 50 µm of anti-apoptotic agents for 24 h were infected with *C. pneumonia* J138 (MOI = 0.2). The infected cells were fixed and stained at 48 hpi. Cells with inclusions larger than 4 µm in diameter were counted as infected ones, and relative infection rates were calculated on the basis of the standard experiment without any inhibitors shown as “−”. Ap-i, C8-i, C9-i, and C3-i indicate cell-permeant inhibitors of Apaf-1 (NS3694), caspase-8 (Z-IETD-FMK), caspase-9 (Z-LEHD-FMK), and caspase-3 (Z-EDVD-FMK), respectively. **b** Bcl-2-overexpressing HeLa cells (*hBCL2* OE) and control cells (Cont) [3] were used for chlamydial infection (MOI = 0.2). Infection rates were calculated at 48 hpi. All data are expressed as mean ± SD from at least three independent experiments. Asterisks denote *p* < 0.05 using Student’s *t* test

The anti-apoptotic activity of chlamydial infection seems to be an advantage for escaping from the host immunosurveillance. It is also possible that this anti-apoptotic environment is also favorable for chlamydial multiplication. To verify this possibility, the susceptibility of host cells to chlamydial infection was assessed by adding anti-apoptotic agents prior to chlamydial infection (Fig. 2a). The cell-permeant irreversible caspase-9 inhibitor (C9-i) decreased the infection rate to nearly half of control, and cell-permeant Apaf-1 inhibitor (Ap-i) conduced a 1.5 times higher infection rate, while caspase-8 and -3 inhibitors (C8-i, C3-i, respectively) showed no modification of infection rates. It has been reported that *C. trachomatis andC. psittaci* induce host apoptosis and that chlamydial infection is inhibited by Bcl-2 overexpression [41]. In contrast, no significant difference was observed in the current study in infection rates or inclusion sizes of *C. pneumoniae* J138 between the HeLa cells overexpressing Bcl-2 and control cells (Fig. 2b).

### Chlamydial infection in Apaf-1- and Caspase-9-deficient cells

**Fig. 3:**
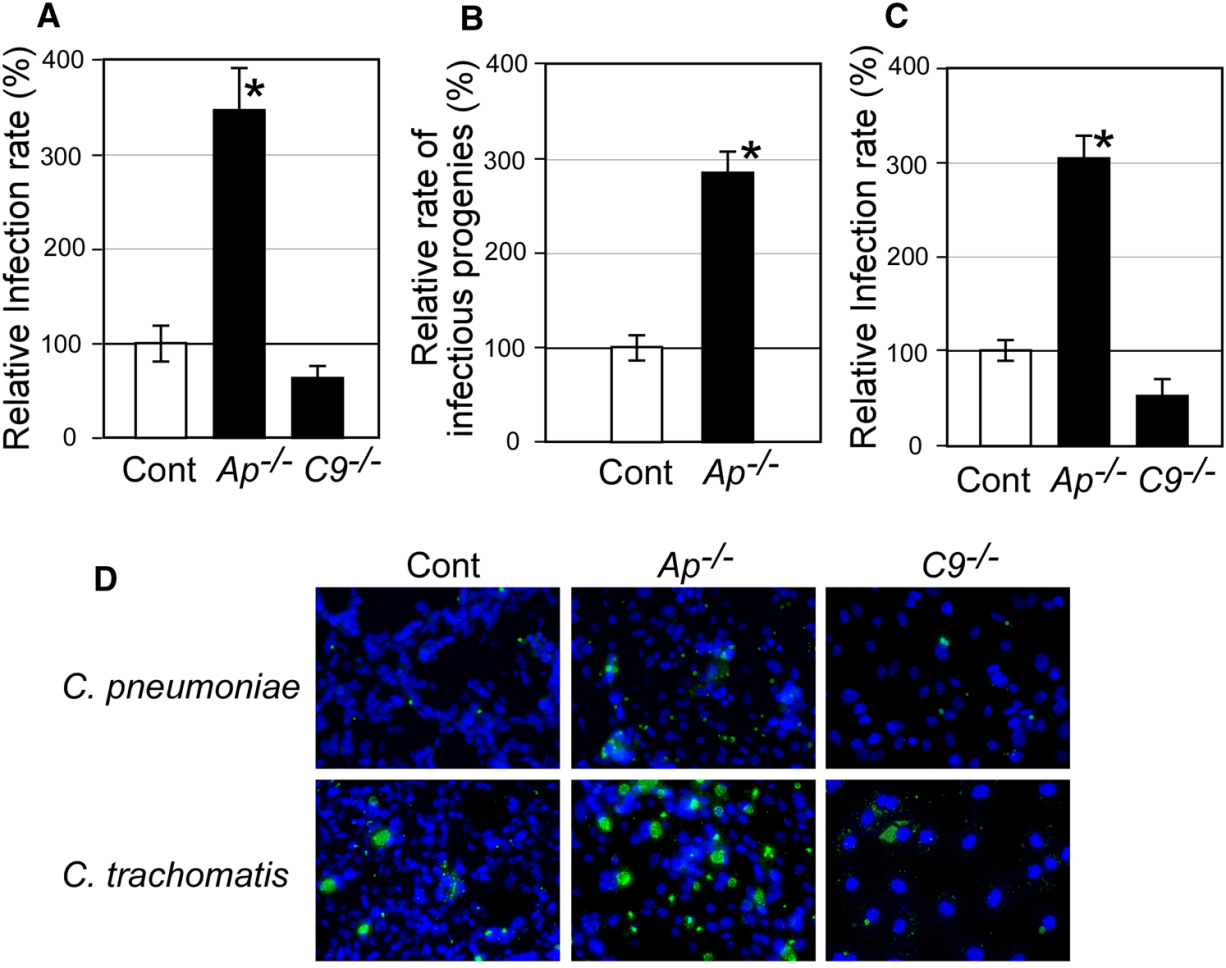
Apaf-1 and caspase-9 show epistatic effects on chlamydial infection. **a** and **d** Apaf-1- and caspase-9-deficient MEFs (indicated as *Ap* ^−*/*−^ and *C9* ^−*/*−^, respectively), and control MEFs (Cont) were subjected to *C. pneumoniae* infection (MOI = 0.2). Samples at 48 hpi were fixed and stained with Hoechst (*blue*) and an anti-chlamydia antibody, RR403, (*green*) and viewed under a fluorescence microscope. Infection rates were calculated using host cells with inclusions larger than 4 µm in diameter. (B) Generation of infectious progenies of *C. pneumoniae* was calculated using Apaf-1-deficient MEFs. After centrifugation of infection medium at 80 hpi, the supernatants were used for re-infection in control MEFs (Cont). The infection rates were measured at 48 hpi. **c** and **d** *C. trachomatis* was used to infect Apaf-1- and caspase-9-deficient MEFs (MOI = 0.2), and control MEFs (Cont). Infection rates were assessed at 20 hpi as for *C. pneumonia*. All data are expressed as mean ± SD from at least three independent experiments. *Asterisks* denote *p* < 0.05 using Student’s *t* test

To confirm the different contributions of Apaf-1 and caspase-9 in chlamydial infection, Apaf-1 and caspase-9 knockout (*apaf*-*1*^−*/*−^ and *caspase*-*9*^−*/*−^, respectively) MEFs were used as host cells for infection. Consistent with the inhibitor treatment results, *C. pneumoniae* infection rates were four times greater in *apaf*-*1*^−*/*−^ MEFs than in controls, and reduced by nearly half in *caspase*-*9*^−*/*−^ MEFs (Fig. 3a). Generation of infectious progenies of *C. pneumoniae* was calculated using *apaf*-*1*^−*/*−^ MEFs. This result was consistent with the infection rate (Fig. 3b). Generation of infectious progenies from *caspase*-*9*^−*/*−^ MEFs was consistent with the infection rate as well (data not shown). Most of phenomena observed here using *C. pneumoniae* J138 were confirmed using *C. pneumoniae* AR39, and very similar data were achieved (data not shown). To test further species specificity, *C. trachomatis* was used for infection of *apaf*-*1*^−*/*−^ and *caspase*-*9*^−*/*−^ MEFs (Fig. 3c). Similar outcomes of infection were observed in both MEFs. In cells infected with both chlamydiae, no morphological changes of host nuclei between infected and non-infected cells were observed (Fig. 3d).

**Fig. 4:**
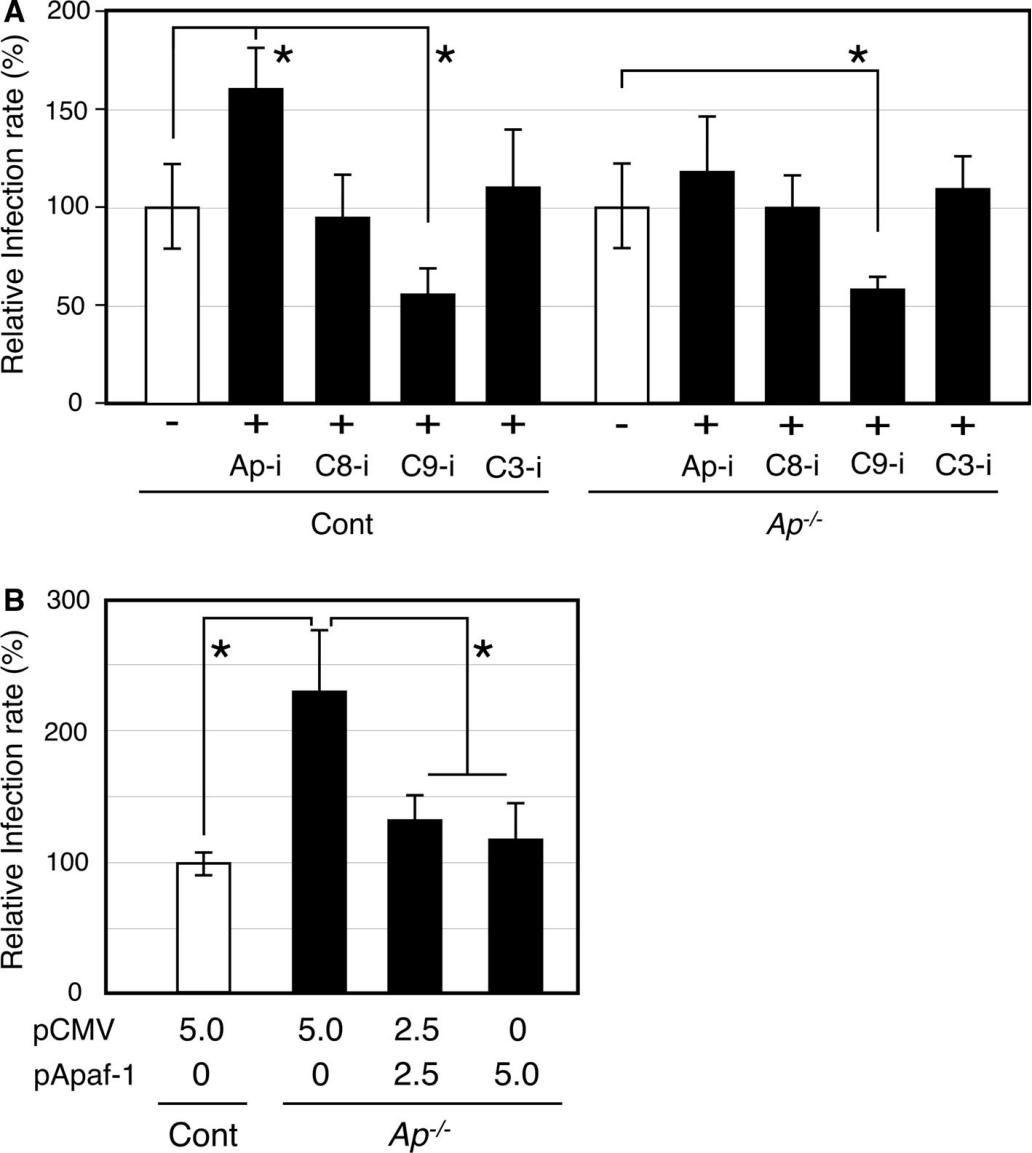
Independent contributions of caspase-9 and Apaf-1 in *C. pneumoniae* infection are confirmed using apoptosis inhibitors and *apaf*-*1* gene complementation. **a** Effects of apoptosis inhibitors at 50 µm were assayed on *C. pneumoniae* infection in MEF cells (*left* panel) and Apaf-1-deficient cells (*right* panel, shown as *Ap* ^−*/*−^). The inhibitors were added from 24 to 48 hpi and infected cells were counted at 48 hpi. Relative infection rates were calculated on the basis of the standard experiment shown as “−”. **b** The pApaf-1 plasmid, consisting of the mouse *apaf*-*1* gene in pCMV-sport 6.1 was transiently transfected into Apaf-1-deficient MEFs. After *C. pneumoniae* infection (MOI = 0.2), infection rates were calculated at 48 hpi. All data are expressed as mean ± SD from at least three independent experiments. *Asterisks* denote *p* < 0.05 using Student’s t test

To clarify whether or not caspase-9 functions independently from Apaf-1, and whether or not Apaf-1 is indeed a target of Ap-i, C9-i and other inhibitors were used during infection of *apaf*-*1*^−*/*−^ MEFs (Fig. 4a). In agreement with the HEp-2 data (Fig. 2), C9-i decreased but Ap-i increased the *C. pneumoniae* infection rates to MEF control cells (Fig. 4a, left panel). When *apaf*-*1*^−*/*−^ MEFs were treated with Ap-i, the incremental increase in the *C. pneumoniae* infection rate was negated; however, C9-i still decreased the infection rate (Fig. 4a, right panel). Complementation assays were performed using the *apaf*-*1* gene and the *apaf*-*1*^−*/*−^ MEFs (Fig. 4b). The results showed that the incremental increase in infection rate was absent, but there was no further decline in the rate at the higher doses of the vector containing the *apaf*-*1* gene.

### Apaf-1-independent caspase-9 activation by chlamydial infection

**Fig. 5:**
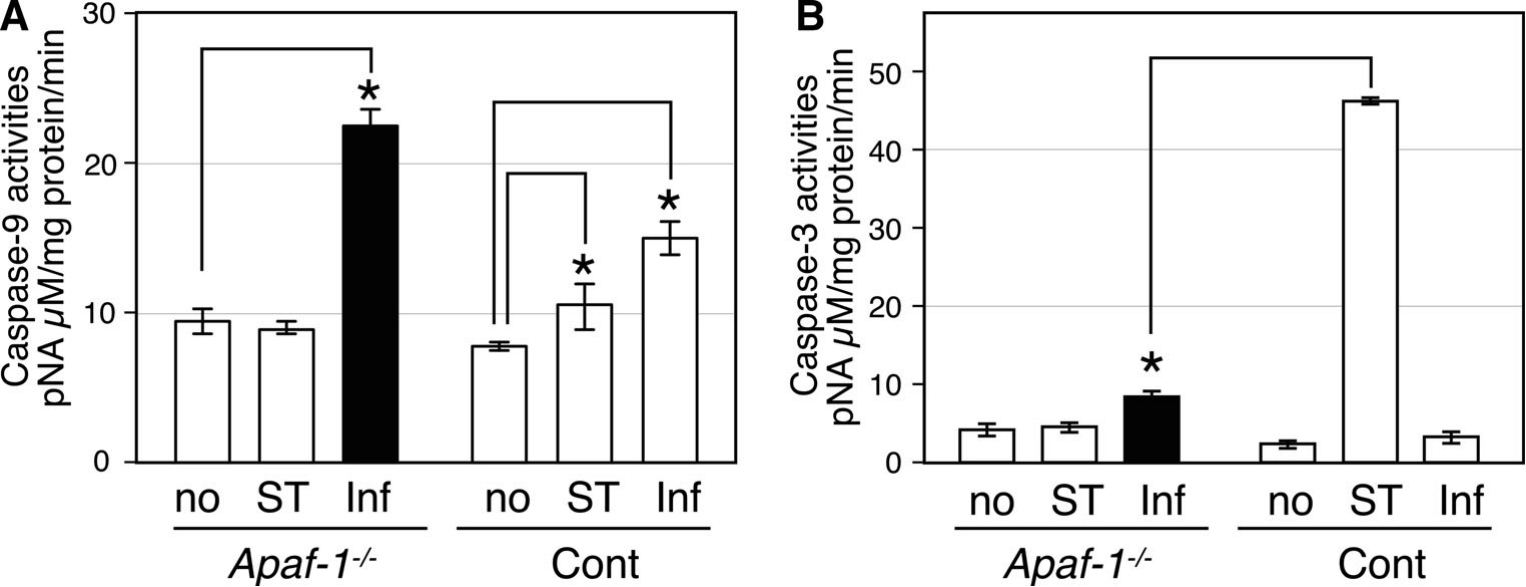
Caspase-9, but not caspase-3, is activated by *C. pneumoniae* infection in a manner independent from Apaf-1. (A and B) Caspase-9 and -3 activities were measured using a colorimetric activity assay. Activities are calculated as released amounts of the chromophore *p*-nitroaniline (*p*NA) per mg protein in cytosolic fractions prepared from *apaf*-*1* ^−/−^ and control MEFs. Mock, STS treatment, and *C. pneumoniae* infection samples are indicated as no, ST and Inf, respectively, in each panel. All data are expressed as mean ± SD from at least three independent experiments. *Asterisks* denote *p* < 0.05 using Student’s *t* test

Caspase-9 is generally activated in apoptosomes that contain Apaf-1, but the C9-i used here, which is an antagonist of caspase-9 self-cleavage, inhibited chlamydial infection. Thus, it is possible that caspase-9 activation is required for chlamydial infection. We evaluated caspase-9 protease activity in host cell cytosolic fractions after infection of *apaf*-*1*^−*/*−^ MEFs (Fig. 5a). As expected, caspase-9 protease activity was increased in the control MEFs but not in *apaf*-*1*^−*/*−^ MEFs with STS treatment. In contrast, *C. pneumoniae* infection significantly increased caspase-9 protease activity in both of *apaf*-*1*^−*/*−^ and control MEFs (Fig. 5a). Generally, caspase-9 activation leads to activation of caspase-3 followed by apoptosis, but apoptosis was not observed after caspase-9 activation by chlamydial infection in *apaf*-*1*^−*/*−^ MEFs (Figs. 2a, 3d). Surprisingly, caspase-3 activity was not significantly increased by chlamydial infection at 48 hpi in the *apaf*-*1*^−*/*−^ and control MEFs, compared to STS treatment (Fig. 5b). Caspase-3 activity in the infected *apaf*-*1*^−*/*−^ MEFs was slightly higher than the background controls, which may be because activated caspase-9 activates caspase-3 when assay samples are prepared in vitro.

**Fig. 6:**
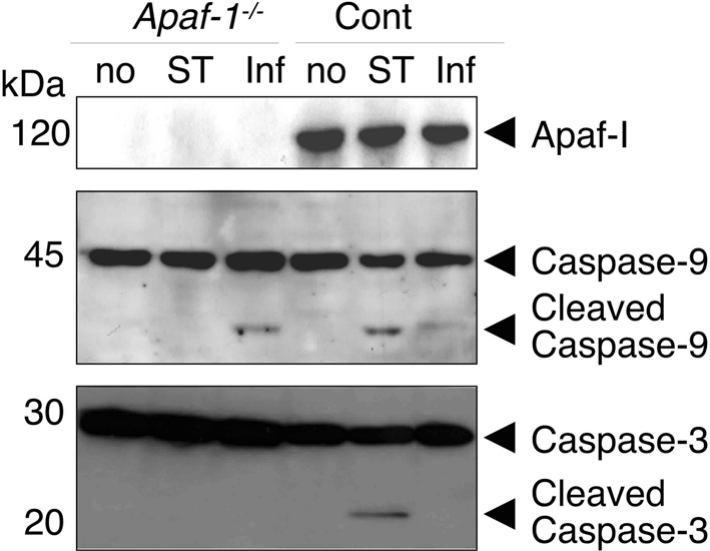
Caspase-9, but not caspase-3, is proteolytically activated by *C. pneumoniae* infection in a manner independent from Apaf-1. Amounts of Apaf-1 protein, caspase-3 and -9 were analyzed by western blot detection using 30 μg total protein prepared from *apaf*-*1* ^−/−^ and control MEFs with STS treatment or chlamydial infection. The data shown here are representative of three independent experiments. Strength of each signal was analyzed using ImageJ

Activation of caspase-3 and -9 was analyzed by western blot. Results were nearly consistent with the activity assays on *apaf*-*1*^−/−^ and control MEFs cytosolic fractions (Fig. 6). The higher levels of activated caspase-9 in the infected *apaf*-*1*^−*/*−^ MEFs compared to the infected control MEFs (approximately 5 times) is due to a higher infection rate in the *apaf*-*1*^−*/*−^ MEFs (Fig. 3a). These results indicate that there is a de novo mechanism for Apaf-1-independent caspase-9 activation during chlamydial infection and that the activated caspase-9 is not engaged in caspase-3 activation for host cell apoptosis.

**Fig. 7:**
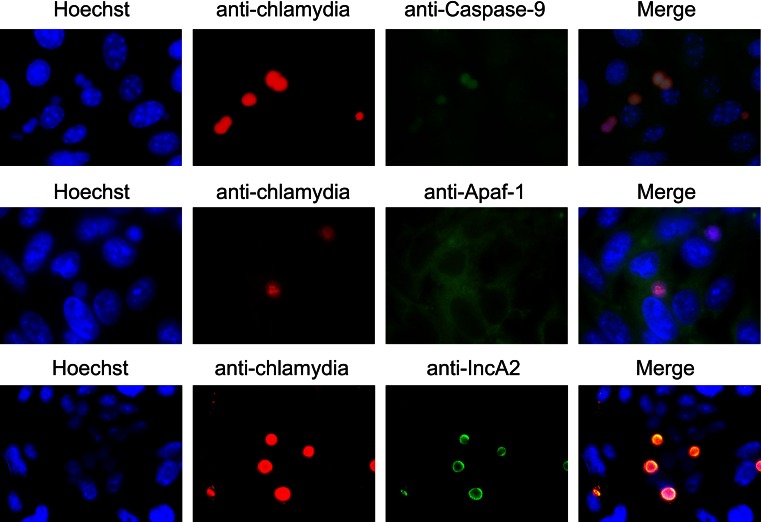
Caspase-9, but not Apaf-1, is co-localized with inclusions of *C. pneumoniae*. *apaf*-*1* ^−/−^ MEFs (*top*) and HEp-2 cells (*middle* and *bottom*) were infected by *C. pneumoniae* and observed at 48 hpi by immunofluorescence staining using Hoechst 33258 (shown as Hoechst in *blue*), an anti-chlamydial specific monoclonal antibody, RR402, (anti-chlamydia in *red*), and anti-caspase-9, anti-Apaf-1 and IncA2 specific antibodies (in *green*). Merged images are shown as Merge in both experiments

To address how caspase-3 is not activated by infection-induced activated caspase-9, we first analyzed expression of genes encoding inhibitors of apoptosis protein IAP1, IAP2, and XIAP. DNA microarray and quantitative RT-PCR at 48 hpi (data not shown) [40], showed that expression of these genes was not increased. However, caspase-9 was co-localized with chlamydial inclusions at 48 hpi by immunofluorescence staining using a caspase-9 antibody with *apaf*-*1*^−/−^ MEFs (Fig. 7) and all inclusions showed the presence of caspase-9. Two additional anti-caspase-9 antibodies purchased from different companies were tested and confirmed this result, and Caspase-9 was not detected in the *caspase*-*9*^−/−^ MEFs (data not shown). Moreover, comparing with localization of chlamydial inclusion membrane protein (IncA2), caspase-9 seemed to localize inside of inclusions. There data suggest the hypothesis that chlamydial infection-mediated repression of apoptosis is at least partially a result of caspase-9 sequestration in inclusions, in which caspase-9 is precluded from its role (or availability) in the host apoptosis cascade. Mechanisms for caspase-9 localization and activation remain as a challenge to comprehend chlamydial developmental cycle.

## Discussion

Modifications of host cell apoptosis by chlamydial infection have been intensively studied. However, the roles of pro- or anti-apoptotic factors in chlamydial infections are not yet elucidated. In this study, we have attempted to clarify host apoptosis regulation by *Chlamydia*, mainly *C. pneumoniae*, using exogenous apoptosis repressors, such as *bcl*-*2* overexpression, chemical apoptosis inhibitors, and gene knockout of apoptotic factors. Based on the results shown here, two hypotheses are proposed regarding an epistatic effect of *apaf*-*1* and *caspase*-*9* on chlamydial infection.

First, both human and mouse cells treated with an apoptosome inhibitor and *apaf*-*1*^−*/*−^ MEFs are more susceptible to chlamydial infections than control cells. The *apaf*-*1* gene could complement the susceptible phenotype. Beyond controversy, Apaf-1 is well-known to oligomerize and activate caspase-9 through a caspase recruitment domain (CARD). Nod1 and Nod2, which also contain the CARDs, were implicated as intracellular sensors that recognize patterns of intracellular pathogens [42, 43], while expression of both Nod1 and Nod2 in HEp-2 cells were not modified by chlamydial infection based on a DNA microarray analysis (data not shown). Thus, it is conceivable that the Apaf-1 functions as a host defense factor against invasion by intracellular pathogens as an inhibitor or sensor as well as a pro-apoptotic agent. In favor of this notion, a non-apoptotic role for Apaf-1 was recently proposed, in which it functions as a DNA damage regulator controlling the checkpoint kinase Chk1 and thus acts as a tumor suppressor [44]. In this case, Apaf-1 may indirectly confine *Chlamydia* to supporting host cell proliferation; however we are proposing another direct response of Apaf-1 against chlamydial infection, as described below.

In an opposite manner, human and mouse cells treated with a caspase-9 inhibitor and *caspase*-*9*^−*/*−^ MEFs are more insusceptible to chlamydial infections than control cells. Interestingly, caspase-9 was activated in *Apaf*-*1*^−*/*−^ MEFs by chlamydial infection, but the activated caspase-9 was disconnected from the caspase cascade that activates caspase-3. Moreover, activated caspase-9 was co-localized with chlamydial inclusions. Taken together, these data suggest that *Chlamydia* require caspase-9 activation for its inclusion maturation and/or multiplication. Therefore, we herein present another model for the repression of apoptosis by chlamydial infection. That is, caspase-9 sequestration by chlamydial infection from the host apoptosis cascade results in apoptosis repression of host cells, and Apaf-1 may compete against chlamydial utilization of caspase-9. This sequestration model is partially similar to those in which phosphorylated Bad was sequestered via 14-3-3 beta to the chlamydial inclusion membrane that contains IncG proteins [30] and a pro-apoptotic effector protein kinase C delta (PKC-δ) was mislocalized according to accumulation of diacylglycerol in the immediate vicinity of chlamydial inclusions [33]. *Chlamydia* might develop this sequestration system to perturb multiple cellular processes of the host, such as rearrangement of the membrane trafficking system for its intracellular multiplication and inhibition of host cell apoptosis for persistent infection.

Despite a well-known role of Apaf-1 in the activation of caspase-9 as the initiation of caspase cascade in a variety of cell models, several reports demonstrated that alternative mechanisms for caspase-9 activation exist independently of Apaf-1 on the basis of certain stimuli, such as the infection of Sendai virus in *apaf*-*1*^−*/*−^ MEFs [45] and UV irradiation in *apaf*-*1*^*fog/fog*^ cells [46]. In *Chlamydia* cases, it is deemed that *Chlamydia* possesses a mechanism for Apaf-1-independent activation of caspase-9 supporting its multiplication in parallel with apoptosis repression by the caspase-9 sequestration. The hypotheses shown here may provide a valuable clue to investigate mechanisms for chlamydial infection causing varied diseases.

## Abbreviations

hpi: Hour post-infection
MOI: Multiplicity of infection
MEF: Mouse embryonic fibroblast
*Ap*^−/−^: Knockout of *apaf*-*1* gene
*C9*^−/−^: Knockout of *caspase*-*9* gene
STS: Staurosporine
CARD: Caspase recruitment domain
IAP: Inhibitor of apoptosis protein

## References

[1] Steller H (1995). Mechanisms and genes of cellular suicide. Science.

[2] Chowdhury I, Tharakan B, Bhat GK (2006). Current concepts in apoptosis: the physiological suicide program revisited. Cell Mol Biol Lett.

[3] Tsujimoto Y (1998). Role of Bcl-2 family proteins in apoptosis: apoptosomes or mitochondria?. Genes Cells.

[4] Kroemer G, Dallaporta B, Resche-Rigon M (1998). The mitochondrial death/life regulator in apoptosis and necrosis. Annu Rev Physiol.

[5] Shimizu S, Narita M, Tsujimoto Y (1999). Bcl-2 family proteins regulate the release of apoptogenic cytochrome c by the mitochondrial channel VDAC. Nature.

[6] Molloy A, Laochumroonvorapong P, Kaplan G (1994). Apoptosis, but not necrosis, of infected monocytes is coupled with killing of intracellular bacillus Calmette-Guerin. J Exp Med.

[7] Gao LY, Kwaik YA (2000). The modulation of host cell apoptosis by intracellular bacterial pathogens. Trends Microbiol.

[8] Grayston JT, Kuo CC, Wang SP, Altman J (1986). A new *Chlamydia psittaci* strain, TWAR, isolated in acute respiratory tract infections. N Engl J Med.

[9] Hahn DL, Dodge RW, Golubjatnikov R (1991). Association of *Chlamydia pneumoniae* (strain TWAR) infection with wheezing, asthmatic bronchitis, and adult-onset asthma. JAMA.

[10] Campbell LA, Kuo CC (2004). *Chlamydia pneumoniae*–an infectious risk factor for atherosclerosis?. Nat Rev Microbiol.

[11] Kinoshita J (2004). Pathogens as a cause of Alzheimer’s disease. Neurobiol Aging.

[12] Miyairi I, Byrne GI (2006). *Chlamydia* and programmed cell death. Curr Opin Microbiol.

[13] Gibellini D, Panaya R, Rumpianesi F (1998). Induction of apoptosis by *Chlamydia psittaci* and *Chlamydia trachomatis* infection in tissue culture cells. Zent Bakteriol.

[14] Ojcius DM, Souque P, Perfettini JL, Dautry-Varsat A (1998). Apoptosis of epithelial cells and macrophages due to infection with the obligate intracellular pathogen *Chlamydia psittaci*. J Immunol.

[15] Perfettini JL, Darville T, Gachelin G, Souque P, Huerre M, Dautry-Varsat A (2000). Effect of *Chlamydia trachomatis* infection and subsequent tumor necrosis factor alpha secretion on apoptosis in the murine genital tract. Infect Immun.

[16] Schoier J, Ollinger K, Kvarnstrom M, Soderlund G, Kihlstrom E (2001). *Chlamydia trachomatis*-induced apoptosis occurs in uninfected McCoy cells late in the developmental cycle and is regulated by the intracellular redox state. Microb Pathog.

[17] Fischer SF, Harlander T, Vier J, Hacker G (2004). Protection against CD95-induced apoptosis by chlamydial infection at a mitochondrial step. Infect Immun.

[18] Rajalingam K, Al-Younes H, Muller A, Meyer TF, Szczepek AJ, Rudel T (2001). Epithelial cells infected with *Chlamydophila pneumoniae* (*Chlamydia pneumoniae*) are resistant to apoptosis. Infect Immun.

[19] Airenne S, Surcel HM, Tuukkanen J, Leinonen M, Saikku P (2002). *Chlamydia pneumoniae* inhibits apoptosis in human epithelial and monocyte cell lines. Scand J Immunol.

[20] Geng Y, Shane RB, Berencsi K, Gonczol E, Zaki MH, Margolis DJ (2000). *Chlamydia pneumoniae* inhibits apoptosis in human peripheral blood mononuclear cells through induction of IL-10. J Immunol.

[21] Fischer SF, Schwarz C, Vier J, Hacker G (2001). Characterization of antiapoptotic activities of *Chlamydia pneumoniae* in human cells. Infect Immun.

[22] Schoier J, Hogdahl M, Soderlund G, Kihlstrom E (2006). *Chlamydia* (*Chlamydophila*) *pneumoniae*-induced cell death in human coronary artery endothelial cells is caspase-independent and accompanied by subcellular translocations of Bax and apoptosis-inducing factor. FEMS Immunol Med Microbiol.

[23] Fan T, Lu H, Hu H, Shi L, McClarty GA, Nance DM (1998). Inhibition of apoptosis in chlamydia-infected cells: blockade of mitochondrial cytochrome c release and caspase activation. J Exp Med.

[24] Fischer SF, Vier J, Kirschnek S, Klos A, Hess S, Ying S (2004). *Chlamydia* inhibit host cell apoptosis by degradation of proapoptotic BH3-only proteins. J Exp Med.

[25] Dong F, Pirbhai M, Xiao Y, Zhong Y, Wu Y, Zhong G (2005). Degradation of the proapoptotic proteins Bik, Puma, and Bim with Bcl-2 domain 3 homology in *Chlamydia trachomatis*-infected cells. Infect Immun.

[26] Ying S, Seiffert BM, Hacker G, Fischer SF (2005). Broad degradation of proapoptotic proteins with the conserved Bcl-2 homology domain 3 during infection with *Chlamydia trachomatis*. Infect Immun.

[27] Pirbhai M, Dong F, Zhong Y, Pan KZ, Zhong G (2006). The secreted protease factor CPAF is responsible for degrading pro-apoptotic BH3-only proteins in *Chlamydia trachomatis*-infected cells. J Biol Chem.

[28] Chen AL, Johnson KA, Lee JK, Sutterlin C, Tan M (2012). CPAF: a Chlamydial protease in search of an authentic substrate. PLoS Pathog.

[29] Snavely EA, Kokes M, Dunn JD, Saka HA, Nguyen BD, Bastidas RJ (2014). Reassessing the role of the secreted protease CPAF in *Chlamydia trachomatis* infection through genetic approaches. Pathog Dis.

[30] Verbeke P, Welter-Stahl L, Ying S, Hansen J, Hacker G, Darville T (2006). Recruitment of BAD by the *Chlamydia trachomatis* vacuole correlates with host-cell survival. PLoS Pathog.

[31] Rajalingam K, Sharma M, Lohmann C, Oswald M, Thieck O, Froelich CJ (2008). Mcl-1 is a key regulator of apoptosis resistance in *Chlamydia trachomatis*-infected cells. PLoS One.

[32] Kun D, Xiang-Lin C, Ming Z, Qi L (2013). *Chlamydia* inhibit host cell apoptosis by inducing Bag-1 via the MAPK/ERK survival pathway. Apoptosis.

[33] Tse SM, Mason D, Botelho RJ, Chiu B, Reyland M, Hanada K (2005). Accumulation of diacylglycerol in the Chlamydia inclusion vacuole: possible role in the inhibition of host cell apoptosis. J Biol Chem.

[34] Wahl C, Maier S, Marre R, Essig A (2003). *Chlamydia pneumoniae* induces the expression of inhibitor of apoptosis 2 (c-IAP2) in a human monocytic cell line by an NF-kappaB-dependent pathway. Int J Med Microbiol.

[35] Rajalingam K, Sharma M, Paland N, Hurwitz R, Thieck O, Oswald M (2006). IAP-IAP complexes required for apoptosis resistance of *C. trachomatis*-infected cells. PLoS Pathog.

[36] Honarpour N, Du C, Richardson JA, Hammer RE, Wang X, Herz J (2000). Adult Apaf-1-deficient mice exhibit male infertility. Dev Biol.

[37] Ohgushi M, Kuroki S, Fukamachi H, O’Reilly LA, Kuida K, Strasser A (2005). Transforming growth factor beta-dependent sequential activation of Smad, Bim, and caspase-9 mediates physiological apoptosis in gastric epithelial cells. Mol Cell Biol.

[38] Rahman MA, Azuma Y, Fukunaga H, Murakami T, Sugi K, Fukushi H (2005). Serotonin and melatonin, neurohormones for homeostasis, as novel inhibitors of infections by the intracellular parasite *Chlamydia*. J Antimicrob Chemother.

[39] Murata M, Azuma Y, Miura K, Rahman MA, Matsutani M, Aoyama M (2007). Chlamydial SET domain protein functions as a histone methyltransferase. Microbiology.

[40] Miura K, Toh H, Hirakawa H, Sugii M, Murata M, Nakai K (2008). Genome-wide analysis of *Chlamydophila pneumoniae* gene expression at the late stage of infection. DNA Res.

[41] Perfettini JL, Reed JC, Israel N, Martinou JC, Dautry-Varsat A, Ojcius DM (2002). Role of Bcl-2 family members in caspase-independent apoptosis during *Chlamydia* infection. Infect Immun.

[42] Werts C, Girardin SE, Philpott DJ (2006). TIR, CARD and PYRIN: three domains for an antimicrobial triad. Cell Death Differ.

[43] Inohara N, Nunez G (2003). NODs: intracellular proteins involved in inflammation and apoptosis. Nat Rev Immunol.

[44] Zermati Y, Mouhamad S, Stergiou L, Besse B, Galluzzi L, Boehrer S (2007). Nonapoptotic role for Apaf-1 in the DNA damage checkpoint. Mol Cell.

[45] Bitzer M, Armeanu S, Prinz F, Ungerechts G, Wybranietz W, Spiegel M (2002). Caspase-8 and Apaf-1-independent caspase-9 activation in Sendai virus-infected cells. J Biol Chem.

[46] Katoh I, Sato S, Fukunishi N, Yoshida H, Imai T, Kurata S (2008). Apaf-1-deficient fog mouse cell apoptosis involves hypo-polarization of the mitochondrial inner membrane, ATP depletion and citrate accumulation. Cell Res.

